# Robotic-assisted transoral removal of a bilateral floor of mouth ranulas

**DOI:** 10.1186/1477-7819-9-78

**Published:** 2011-07-18

**Authors:** Rohan R Walvekar, Geoffrey Peters, Elliot Hardy, Leonard Alsfeld, Frederick W Stromeyer, Dwayne Anderson, Michael DiLeo

**Affiliations:** 1 Department of Otolaryngology Head Neck Surgery, LSU Health Sciences Center, New Orleans, LA, USA; 2 Pathology Group of Louisiana, Baton Rouge, LA, USA; 3 Department of Radiology, Our Lady of the Lake Regional Medical Center, Baton Rouge, LA, USA

## Abstract

**Objective:**

To describe the management of bilateral oral ranulas with the use of the da Vinci Si Surgical System and discuss advantages and disadvantages over traditional transoral resection.

**Study Design:**

Case Report and Review of Literature.

**Results:**

A 47 year old woman presented to our service with an obvious right floor of mouth swelling. Clinical evaluation and computerized tomography scan confirmed a large floor of mouth ranula on the right and an incidental asymptomatic early ranula of the left sublingual gland. After obtaining an informed consent, the patient underwent a right transoral robotic-assisted transoral excision of the ranula and sublingual gland with identification and dissection of the submandibular duct and lingual nerve. The patient had an excellent outcome with no evidence of lingual nerve paresis and a return to oral intake on the first postoperative day. Subsequently, the patient underwent an elective transoral robotic-assisted excision of the incidental ranula on the left sublingual gland.

**Conclusion:**

We describe the first robotic-assisted excision of bilateral oral ranulas in current literature. The use of the da Vinci system provides excellent visualization, magnification, and dexterity for transoral surgical management of ranulas with preservation of the lingual nerve and Wharton's duct with good functional outcomes. However, the use of the robotic system for anterior floor of mouth surgery in terms of improved surgical outcomes as compared to traditional transoral surgery, long-term recurrence rates, and cost effectiveness needs further validation.

## Introduction

The ranula is an extravasation mucocele that arises from the sublingual gland, either from a ruptured main salivary duct or from ruptured acini following obstruction[1]. In a study of 580 ranulas, most patients with oral ranula presented with a gradually increasing round or oval, fluctuant swelling of the floor of the mouth. Majority of ranula ranged between 2 to 3 cm in size. Ranulas most commonly occurred as a unilateral swelling but were found to be bilateral in 1.5% cases (9/580). The occurrence as bilateral and simultaneous ranulas was even more uncommon (0.5%; 3/580), as seen in our case [2]. A more advanced presentation of ranula is the plunging ranula that is an extension of the oral ranula into the neck along the deep lobe of the submandibular gland between the mylohyoid and hyoglossus muscles or through congenital dehiscence in the mylohyoid muscle [3,4].

The therapeutic options for oral and plunging ranulas are aimed at either surgical excision of the lesion or attempts at inducing fibrosis and scarring that would eliminate the formation of the ranula [1,3,5]. These interventions can range from simple incision, marsupialization with or without packing, excision of the ranula with or without the sublingual gland, laser vaporization and the use of sclerosing agent OK-432[1,4]. Excision of the ranula with the associated sublingual gland is associated with the best outcomes with lowest recurrence rates [1-3,5]. Usually, this can be accomplished via a transoral route.

The challenges of ranula excision and of floor mouth surgery involve the identification and preservation of the submandibular duct (Wharton's duct), lingual nerve and its terminal branches, and excision of the entire sublingual gland transorally. Transoral excision can be challenging especially when faced with difficult anatomy. It also requires an experienced assistant. We present a novel modification to the traditional transoral resection using the da Vinci Si Robotic Surgical System. The use of the da Vinci robotic system for tumors of the head and neck is a new technological advance. Current validated indications for the use of the robot in head and neck surgery include the management of benign and malignant tumors of the tonsil and base of the tongue and for distant access trans-axillary surgery for removal of the lesions within the thyroid gland. The da Vinci robotic system has also been reported to be useful for the surgical management of hypopharyneal, laryngeal and parapharyngeal space tumors [6-11]. This is the first description of the use of surgical robot for management of oral floor of mouth ranulas. We present our experience and discuss advantages and disadvantages of the da Vinci robotic system in managing anterior floor of mouth lesions.

## Case Report

**Figure 1:**
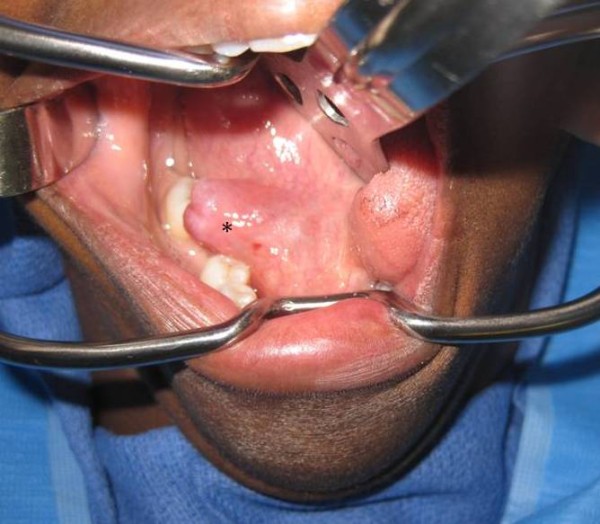
Clinical picture showing a right floor of mouth ranula (* indicates the lesion).

**Figure 2:**
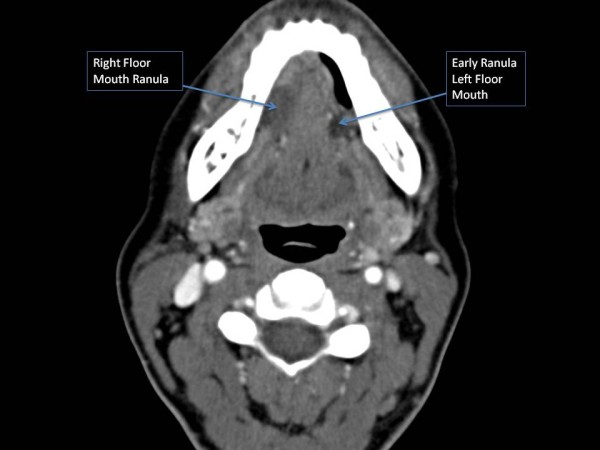
**An axial contrast enhanced CT image depicting bilateral floor of mouth ranula**.

A 47-year old woman was referred to the Head and Neck Center at Our Lady of the Lake Regional Medical Center in Baton Rouge, LA and to the Department of Otolaryngology Head & Neck Surgery, Louisiana State University Health Sciences Center, New Orleans, LA to be evaluated for a right floor of mouth swelling (Figure 1). The swelling was associated with progressive discomfort in speech. There were no symptoms suggestive of an infective or obstructive process within the submandibular system such as pain, fever, or an association of the swelling with meals. At this time, a computerized tomography (CT) scan confirmed a right oral ranula that measured 2.4 × 1.6 × 1.0 cm in size (Figure 2). In addition, an incidental ranula of the left floor or mouth was identified. After discussing the surgical options with the patient that included marsupialization, resection of the ranula, and resection of the ranula and sublingual gland, the patient decided to opt for the surgical removal of the right-sided ranula that was symptomatic with the ipsilateral sublingual gland. The informed consent also included the use *da Vinci Si *Surgical System to optimize surgical exposure and access. The patient underwent an uneventful procedure with identification of the lingual nerve and submandibular duct using the robotic unit. The patient had an uneventful post-operative course without any evidence of lingual nerve paresis and a return to oral intake on the first postoperative day. Consequently, the patient underwent an elective resection of the left-sided early ranula and excision of the sublingual gland. This was accomplished with the da Vinci Si surgical robot as well without complications. The patient did not have any evidence of submandibular duct or lingual nerve injury as evidenced by the patient's symptoms and post-operative evaluations. Final histopathology was benign sublingual gland ranula on both sides.

### Surgical Approach

**Figure 3:**
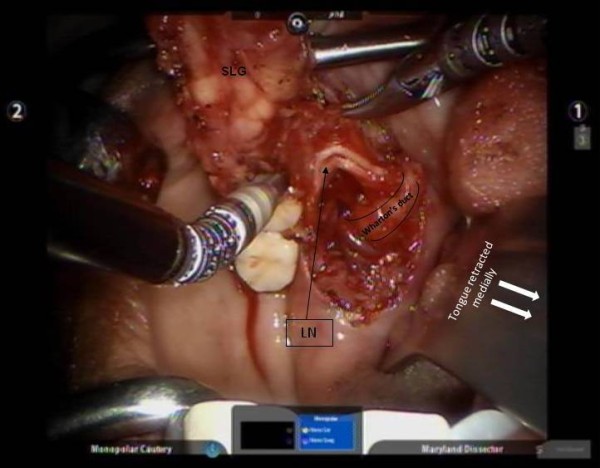
**Dissection of the left sublingual gland (SLG) with identification of the terminal branches of the lingual nerve and delineation of the Wharton's duct**.

The transoral resection of the ranula was performed using the da Vinci Si Surgical System. The oral cavity and surgical site were exposed using a self-retaining retractor (Jennings's mouth gag) and a Sweetheart tongue retractor. The robotic arms of the da Vinci Si-System were placed into position in the patient's mouth, while the surgeon controlled the instruments from the control console within the room. The robotic arm controlled by the surgeon's left hand contained a 5 mm Maryland dissecting forceps, while the arm controlled by his right hand contained a 5 mm monopolar cautery spatula. These instruments were interchanged as dictated by the need for surgical dissection. Retraction of the tongue and suction were provided by the first assistant at the head end of the patient as described for transoral robotic surgery. The initial incision was made using cautery in the floor of the mouth. The sublingual gland was meticulously dissected and separated from the lingual nerve and the Wharton duct (Figure 3). The lingual nerve was dissected along its length to confirm identification of terminal branches and to separate it from the salivary duct. The sublingual gland and ranula were excised. The mucosa of the floor mouth was also approximated with four interrupted 3-0 absorbable stitches. The total procedure times were 44 and 59 minutes for the right and left side, respectively. The time required for exposure including robot "docking time" was 6 and 8 minutes, respectively. The procedure times for the right and left side were 38 minutes and 51 minutes, respectively. There were no major intraoperative complications. The patient tolerated the procedure well and was discharged home the same day in both instances.

## Discussion

Transoral resection of the ranula with the involved sublingual gland provides the best outcomes for ranula surgery with the least recurrence rates [1-3,5]. In a study of 606 procedures in 571 patients, Zhao et al reported the most common complications associated with transoral ranula surgery included recurrence of the lesion (34.6%), sensory deficits of the tongue associated with lingual nerve injury (29.3%), and damage to the Wharton's duct (14.6%) [12]. These complication rates can be reduced or minimized by improving visualization, magnification, illumination, and reducing intraoperative hemorrhage. Guerrissi and Taborda reported their experience with endoscopy assisted transoral submandibular gland excision. In this article, the authors found that the use of the endoscope allowed improved illumination, visualization, and magnification of the operative field and also provided better visual access to the vascular pole of the submandibular gland [13]. Lai et al (2009) in their study describing the use of carbon dioxide laser for the management of oral ranulas, suggested that their improved outcomes and early recovery rates were influenced by the use of the laser which allowed precise cutting, minimal thermal damage, better visualization of the operative site due to reduced intraoperative hemorrhage [4].

In a similar fashion, we found that the use of the robotic unit provides certain advantages while performing transoral floor of mouth surgery. First, the da Vinci Si Surgical System incorporates two separate high definition optical channels that merge to produce a high-definition, three-dimensional image at the surgeon's console [14]. Second, the magnification and dexterity provided by the robot in the confined space of the oral cavity allow precise dissection and preservation of delicate floor of mouth structures namely, the lingual nerve and Wharton's duct. Third, the 5 mm wristed instruments that have 6 degrees of articulation that facilitate surgical dissection and delicate handling of floor of mouth structures. Fourth, the surgeon and assistants can work in tandem as all surgical steps are visualized by the surgical team and the operating room staff. This not only improves surgical efficiency but also serves as an excellent teaching tool for residents, medical students, and operating room staff. The camera in the docked position provides a direct view of the floor of the mouth, medial aspect of the floor of the mouth, and the lingual surface of the mandible. This view can be difficult to obtain in routine transoral surgery based on the shape of the mandible, size of the teeth, extent of pathology, tongue size, and availability of adequate surgical assistance. Due to the above mentioned factors, the authors experience suggests that the use of the robotic system also makes transoral floor of mouth dissection more predictable due to a stable operating view with reduced effort due to improved exposure, dexterity, and comfortable surgeon position at the surgeon console.

The da Vinci robotic system is currently used in the head and neck for the management of tumors of the tonsil and tongue base and the thyroid gland [6,8,11]. We recently reported the first description of the use of the da Vinci Si Surgical system to facilitate a transoral removal of a submandibular gland megalith[15]. We have found the robotic system to similarly provide significant advantages in surgery of the anterior floor mouth.

## Conclusion

The use of the da Vinci system provides excellent visualization, magnification, and dexterity for transoral surgical management of ranulas with preservation of the lingual nerve and Wharton's duct with good functional outcomes. However, the use of the robotic system for anterior floor of mouth surgery in terms of improved surgical outcomes as compared to traditional transoral surgery, long-term recurrence rates, and cost effectiveness needs further validation.

## Competing interests

The authors declare that they have no competing interests.

## Authors' contributions

**RW: **Performed the procedure, wrote the manuscript the First Author and is the Corresponding author for the manuscript. **GP: **Assisted in the procedure, manuscript preparation and editorial review. **EH: **Helped in literature review, formatting images, and data collection. **LA: **Editorial review and helped in literature search. **FS: **Pathologist, provided pathology inputs and diagnosis, editorial review of manuscript. **DA: **Radiologist, reviewed images and provided images, editorial review. **MD: **editorial review, manuscript critical review. All authors read and approved the final manuscript.
